# Correlation between C-Reactive Protein to Albumin Ratio and Disease Activity in Patients with Axial Spondyloarthritis

**DOI:** 10.1155/2021/6642486

**Published:** 2021-06-12

**Authors:** Zheng Zhong, Yukai Huang, Yuqi Liu, Junming Chen, Meng Liu, Qidang Huang, Shaoling Zheng, Xin Guo, Weiming Deng, Tianwang Li

**Affiliations:** ^1^Department of Rheumatology and Immunology, Guangdong Second Provincial General Hospital, Guangzhou, Guangdong 510317, China; ^2^The Second School of Clinical Medicine, Southern Medical University, Guangzhou, Guangdong 510515, China

## Abstract

**Background:**

The C-reactive protein (CRP) to albumin (ALB) ratio (CAR) has emerged as a novel inflammatory biomarker. This study was designed to investigate the role of CAR in the disease activity of axial spondyloarthritis (axSpA).

**Methods:**

A total of 241 patients and 61 healthy controls were retrospectively enrolled in this study. AxSpA patients were further divided into the inactive group (*n* = 176) and active group (*n* = 65) according to Bath Ankylosing Spondylitis Disease Activity Index (BASDAI) cutoff value of 4. Laboratory data and clinical assessment indices were recorded. Spearman's correlation analysis, receiver operation characteristic (ROC) curve analysis, and binary logistic regression analysis were performed.

**Results:**

In axSpA patients, CAR was significantly higher than the healthy group (*P* < 0.001). Similarly, axSpA patients in the active group had higher CAR than the inactive group (*P* < 0.001). Besides, CAR was positively correlated with erythrocyte sedimentation rate (ESR) (*r* = 0.704, *P* < 0.001), CRP (*r* = 0.996, *P* < 0.001), BASDAI (*r* = 0.329, *P* < 0.001), and Bath Ankylosing Spondylitis Functional Index (BASFI) (*r* = 0.330, *P* < 0.001). ROC curve analysis suggested that the area under the curve (AUC) of CAR for axSpA of the active group was 0.701, which was higher than that of CRP and ESR. The optimal cutoff point of CAR for axSpA of the active group was 0.3644, with a sensitivity and specificity of 58.5% and 79.0%. Binary logistic analysis results revealed that CAR was an independent predictive factor for axSpA disease activity (odds ratio = 4.673, 95% CI: 1.423-15.348, *P* = 0.011).

**Conclusions:**

CAR was increased in axSpA and axSpA of the active group. CAR may be a novel and reliable indicator for axSpA disease activity.

## 1. Introduction

Axial spondyloarthritis (axSpA) is a common inflammatory and autoimmune disease characterized by chronic back pain and morning stiffness, which is divided into nonradiographic and radiographic disease according to radiographic structural damage in the sacroiliac joints [1]. The prevalence of axSpA varies from 0.32% to 1.4% in different countries with a female to male ratio of about 1 : 2-3 [2]. Although the exact etiology of axSpA still remains obscure, inflammation is reported to be involved in the pathogenesis and development of axSpA, contributing to the deformity and disability in the late disease stage [3, 4]. Therefore, it is crucial to monitor the inflammation state and disease activity of axSpA. Erythrocyte sedimentation rate (ESR) and C-reactive protein (CRP) are the most widely used inflammatory indicators and increased in active disease status, but the specificity and sensitivity of them are limited and they only reflect the short-term inflammatory activity [5–7].

Many studies have shown that the whole blood components including neutrophils, lymphocytes, monocytes, and platelets were correlated with systemic inflammation [8–10]. Neutrophil to lymphocyte ratio (NLR), monocyte to lymphocyte ratio (MLR), and platelet to lymphocyte ratio (PLR) have been identified as new inflammatory markers and reported to be higher in axSpA patients and related to disease activity [11–13]. However, the results are contradictory with other studies [14, 15]. Albumin (ALB) is a plasma protein and functions in maintaining colloid pressure and transporting free fatty acids, drug metabolites, and bilirubin, whose synthesis is reported to be affected by inflammation [16]. In recent years, CRP to ALB ratio (CAR) has emerged as a novel inflammatory indicator to evaluate inflammation and predict the prognosis of several cancers, such as Ewing sarcoma, esophageal cancer, and colorectal cancer [17, 18]. Some studies have demonstrated the correlations of CAR with disease activity of inflammatory diseases, such as Crohn's disease and rheumatoid arthritis [19, 20]. However, the role of CAR in axSpA has not been investigated yet. Hence, this study was designed to investigate the level of CAR and the relationship between CAR and disease activity in axSpA patients.

## 2. Materials and Methods

### 2.1. Subjects

We performed a retrospective, cross-sectional study in Guangdong Second Provincial General Hospital from December 2015 to August 2019. A total of 241 axSpA patients and 61 healthy controls were enrolled [21]. The diagnosis met the 2009 Assessment of SpondyloArthritis International Society (ASAS) Classification Criteria. Exclusion criteria were as follows: concomitant infection, cardiovascular diseases, diabetes mellitus, renal and hepatic dysfunction, liver cirrhosis, malignancy, tuberculosis, pregnancy, malnutrition, and other inflammatory and autoimmune diseases. This study was approved by the ethics committee office of Guangdong Second Provincial General Hospital (2017-FSMY-009).

### 2.2. Demographic and Laboratory Data

Demographic and laboratory data including age, gender, CRP, ESR, lymphocyte, monocyte, neutrophil, platelet, ALB, and medications were recorded. CAR, NLR, MLR, and PLR were calculated. All peripheral venous blood samples were drawn from 6 to 8 am after overnight fasting and examined for the routine blood test and biochemical test within 2 hours after sample collection. Bath Ankylosing Spondylitis Disease Activity Index (BASDAI) [22, 23] and Bath Ankylosing Spondylitis Functional Index (BASFI) [24] were also collected. Patients were assigned into two groups based on a cutoff value of 4, with BASDAI < 4 in the inactive group and BASDAI ≥ 4 in the active group.

### 2.3. Statistical Analysis

SPSS 19.0, GraphPad Prism 6.0, and MedCalc software were used to carry out statistical analyses. Continuous variables were expressed as mean ± standard deviation, and categorical variables were presented as percentages or numbers. Comparisons of continuous variables were performed by Student's *t*-test or Mann–Whitney *U* test, while categorical variables were analyzed by chi-square test. The associations of CAR with inflammatory indicators and disease activity were assessed by Spearman's correlation analysis. Receiver operating characteristic (ROC) curves were performed, and the areas under the ROC curves (AUCs) were calculated to assess the utility of variables to discriminate the disease activity. In order to explore the predictive factors of axSpA disease activity, logistic regression analysis was performed. *P* value less than 0.05 was considered statistically significant.

## 3. Results

### 3.1. Demographic and Laboratory Data of the Subjects

Demographic and laboratory data of healthy controls and axSpA patients were summarized (Table 1). There were no statistical differences with regard to age (*P* = 0.051) and sex (*P* = 0.174) between healthy controls and axSpA patients. CAR, NLR, PLR, MLR, ESR, and CRP were significantly higher in axSpA patients than those in healthy controls (*P* < 0.001, respectively, except for PLR, *P* = 0.018), while ALB was significantly lower in axSpA patients (*P* < 0.001).

### 3.2. Comparisons of Variables between Active Group and Inactive Group of axSpA

According to the BASDAI, the inactive group included 176 patients and the active group included 65 patients. No statistical differences were detected in age (*P* = 0.255) and gender (*P* = 0.982) between the active group and the inactive group. In comparison with the inactive group, CAR, ESR, CRP, BASDAI, and BASFI were significantly higher in the active group, while ALB was lower (*P* < 0.001, respectively) (Table 2).

### 3.3. Correlations of Variables with ESR, CRP, BASDAI, and BASFI

**Figure 1:**
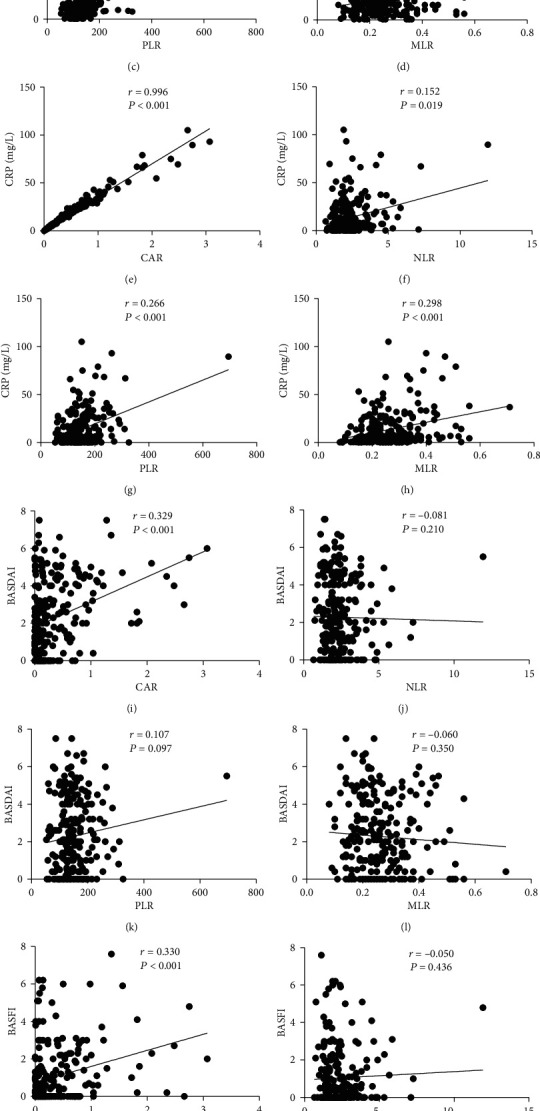
Correlations of CAR, NLR, PLR, MLR with ESR, CRP, BASDAI, and BASFI in axSpA patients.

CAR, NLR, PLR, and MLR were all positively correlated with ESR level (*r* = 0.704, *P* < 0.001; *r* = 0.204, *P* = 0.001; *r* = 0.377, *P* < 0.001; and *r* = 0.185, *P* = 0.004, respectively) and CRP level (*r* = 0.996, *P* < 0.001; *r* = 0.152, *P* = 0.019; *r* = 0.266, *P* < 0.001; and *r* = 0.298, *P* < 0.001, respectively) (Figure 1). CAR, CRP, and ESR were positively associated with BASDAI (*r* = 0.329, *P* < 0.001; *r* = 0.314, *P* < 0.001; and *r* = 0.322, *P* < 0.001, respectively) and BASFI (*r* = 0.330, *P* < 0.001; *r* = 0.319, *P* < 0.001; and *r* = 0.303, *P* < 0.001, respectively), while no correlations were found between NLR, PLR, MLR, BASDAI, and BASFI (Figure 1 and Figure S1).

### 3.4. Diagnostic Values of Variables for Discriminating axSpA of Active Group from Inactive Group

**Figure 2:**
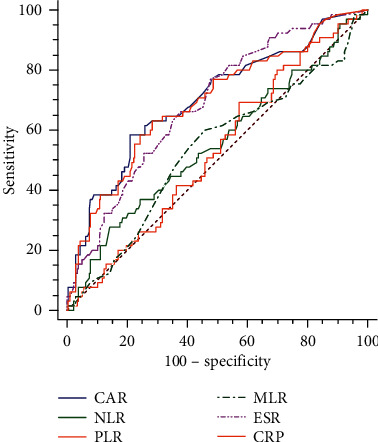
ROC curve analysis of the variables for distinguishing axSpA disease activity.

ROC curves were depicted to assess the values of CAR, NLR, PLR, MLR, ESR, and CRP for discriminating axSpA of active group from inactive group (Figure 2). CAR yielded the highest AUC (0.701, 95% CI: 0.638-0.758) than NLR (0.550, 95% CI: 0.485-0.614), PLR (0.528, 95% CI: 0.463-0.592), MLR (0.532, 95% CI: 0.466-0.596), ESR (0.685, 95% CI: 0.622-0.743), and CRP (0.691, 95% CI: 0.629-0.749) (Table 3). The optimal cutoff point of CAR for axSpA of active group was 0.3644, with a sensitivity and specificity of 58.5% and 79.0%.

### 3.5. Binary Logistic Regression Analysis of Independent Predictive Factors for axSpA Disease Activity

Besides, in order to analyze the independent predictive factors for axSpA disease activity, binary logistic regression analysis was performed. After univariate regression analysis, CAR, NLR, PLR, MLR, ESR, and CRP were analyzed in multivariate regression analysis. The result indicated that only CAR was an independent predictive factor for axSpA disease activity (odds ratio = 4.673, 95% CI: 1.423-15.348, *P* = 0.011) (Table 4).

## 4. Discussion

In this study, CAR was found to be increased in axSpA and axSpA of the active group. CAR showed positive correlations with BASDAI and BASFI. ROC curve analysis revealed that CAR yielded the best value in distinguishing the active group from the inactive group. Besides, logistic regression analysis revealed that CAR was an independent predictive factor for axSpA disease activity.

AxSpA is a common inflammatory and autoimmune disease with inflammation of axial skeleton [25]. Increasing evidence shows that neutrophils, platelets, monocytes, and lymphocytes are involved in the inflammation and thus the combinations of these parameters, NLR, PLR, and MLR, have emerged as novel inflammatory indicators in many inflammatory diseases, including axSpA [13, 26, 27]. Previous studies found that NLR, PLR, and MLR were significantly higher in axSpA patients compared with healthy controls [13]. Consistently, the present study showed increased NLR, PLR, and MLR in axSpA patients. However, the results about the relationship between NLR, PLR, MLR, and inflammation and disease activity of axSpA remain conflicting. Enginar et al. reported that PLR and NLR were associated with ESR, CRP, and disease activity [28], while Bozan et al. found no correlations between PLR, MLR, and BASDAI in axSpA patients [29]. Yang et al. revealed positive correlations of MLR with traditional inflammatory indicators [14], while Liu et al. showed that there was only a positive correlation of MLR with CRP but not with ESR [15]. In this study, correlations were only found between NLR, PLR, MLR, CRP, and ESR but not clinical assessment indices BASDAI or BASFI. Therefore, it is necessary to find more reliable biomarkers evaluating the disease activity of axSpA.

CRP is synthesized by the liver and reflects systemic inflammation, which is widely used as an inflammatory indicator in the clinic [30]. ALB is recognized as an indicator for nutrition status, which is decreased in serious malnutrition, chronic inflammatory, and autoimmune diseases [31]. Therefore, CAR, integrating the effects of CRP and ALB, could be a strong indicator for assessing inflammation. Recently, CAR has emerged as a novel inflammatory biomarker for evaluating disease activity in some inflammatory diseases, such as rheumatoid arthritis [19]. However, the relationship between CAR and axSpA was rarely investigated. To the best of our knowledge, this study is the first to investigate the role of CAR in axSpA. Our results showed that CAR was significantly increased in axSpA patients and strongly associated with ESR and CRP, indicating that CAR could be a reliable inflammatory biomarker in axSpA patients. Besides, significantly higher CAR was detected in the active group compared to the inactive group, and positive correlations were found between CAR and BASDAI and BASFI. The AUC of CAR was higher than other biomarkers (NLR, PLR, MLR, ESR, and CRP) in discriminating axSpA patients of the active group from inactive group, reflecting that CAR may serve as a superior indicator in assessing disease activity in axSpA patients. It was reported that synthesis of CRP was induced by interleukin-6 (IL-6), and synthesis of ALB was inhibited by tumor necrosis factor (TNF) and IL-6 which were proinflammatory [32, 33]. Previous studies have demonstrated that TNF and IL-6 are involved in the initiation and maintenance of inflammation of axSpA [34], which may account for the associations between increased CAR and inflammation and disease activity of axSpA. Furthermore, several studies have revealed that CAR had prognostic value in cancers [35]. Similarly, this study showed that CAR was an independent predictive factor of axSpA disease activity, indicating that increased CAR may predict active disease activity in axSpA.

Some limitations were included in this study. One limitation was that it was a single-center study with a relatively small sample size, so the results may not be extrapolated to the general population. Second, given that our study was a retrospective study, selection bias and recall bias were unavoidable. Third, some data about radiological scores and disease duration were missing, so we could not assess the effect of radiological changes and disease duration on the results. Thus, the findings of our study need further validation by more large-sample, multicenter, and prospective studies.

In conclusion, CAR was increased in axSpA and axSpA of the active group. CAR was an independent predictive factor for axSpA disease activity. CAR may be a novel and reliable biomarker for assessing the disease activity of axSpA patients.

## Figures and Tables

**Table 1:** Demographic and laboratory data of the subjects.

	axSpA (*n* = 241)	Control (*n* = 61)	Reference interval	*P* value
Age (years)	29.61 ± 7.92	31.15 ± 6.05		0.051
Sex (male/female)	200/41	46/15		0.174
Neutrophils (×10^9^/L)	4.42 ± 1.36	3.72 ± 1.39	1.8-6.3	<0.001
Lymphocytes (×10^9^/L)	2.04 ± 0.63	2.15 ± 0.70	1.1-3.2	0.300
Monocytes (×10^9^/L)	0.51 ± 0.18	0.38 ± 0.14	0.1-0.6	<0.001
Platelets (×10^9^/L)	278.28 ± 76.42	251.57 ± 45.24	125-350	0.032
ALB (g/L)	41.73 ± 5.45	46.11 ± 3.73	35-50	<0.001
CAR	0.36 ± 0.51	0.06 ± 0.03		<0.001
NLR	2.39 ± 1.21	1.83 ± 0.73		<0.001
PLR	147.62 ± 61.39	128.88 ± 46.96		0.018
MLR	0.26 ± 0.10	0.19 ± 0.06		<0.001
ESR (mm/h)	25.72 ± 25.08	8.06 ± 6.03	0-20	<0.001
CRP (mg/L)	13.50 ± 17.92	2.66 ± 1.49	0-8	<0.001
Medications (*n* (%))				
NSAIDs	128 (53.11)			
DMARDs	96 (39.83)			
Biologicals	46 (19.09)			

ALB: albumin; CAR: C-reactive protein to albumin ratio; NLR: neutrophil-lymphocyte ratio; PLR: platelet-lymphocyte ratio; MLR: monocyte-lymphocyte ratio; CRP: C-reactive protein; ESR: erythrocyte sedimentation rate; NSAIDs: nonsteroidal anti-inflammatory drugs; DMARDs: disease modifying anti-rheumatic drugs.

**Table 2:** Comparisons of the variables between axSpA of the active group and inactive group.

	Inactive group (*n* = 176)	Active group (*n* = 65)	*P* value
Age (years)	29.22 ± 7.49	30.66 ± 8.95	0.255
Sex (male/female)	146/30	54/11	0.982
Neutrophils (×10^9^/L)	4.37 ± 1.32	4.55 ± 1.47	0.415
Lymphocytes (×10^9^/L)	1.98 ± 0.61	2.18 ± 0.66	0.022
Monocytes (×10^9^/L)	0.50 ± 0.18	0.54 ± 0.20	0.192
Platelets (×10^9^/L)	266.88 ± 68.77	309.15 ± 87.41	<0.001
ALB (g/L)	42.83 ± 5.08	38.77 ± 5.37	<0.001
CAR	0.26 ± 0.39	0.62 ± 0.69	<0.001
NLR	2.40 ± 1.09	2.35 ± 1.51	0.230
PLR	145.09 ± 52.31	154.49 ± 81.15	0.504
MLR	0.26 ± 0.10	0.26 ± 0.10	0.452
ESR (mm/h)	21.51 ± 20.89	37.13 ± 31.39	<0.001
CRP (mg/L)	10.36 ± 15.13	22.00 ± 21.86	<0.001
BASDAI	1.28 ± 1.17	4.98 ± 0.84	<0.001
BASFI	0.58 ± 0.98	2.31 ± 1.98	<0.001

ALB: albumin; CAR: C-reactive protein to albumin ratio; NLR: neutrophil-lymphocyte ratio; PLR: platelet-lymphocyte ratio; MLR: monocyte-lymphocyte ratio; CRP: C-reactive protein; ESR: erythrocyte sedimentation rate; BASDAI: Bath Ankylosing Spondylitis Disease Activity Index; BASFI: Bath Ankylosing Spondylitis Functional Index.

**Table 3:** Diagnostic values of variables for discriminating axSpA of the active group from the inactive group.

	AUC	95% CI	Optimal cutoff point	Specificity	Sensitivity
CAR	0.701	0.638-0.758	0.3644	79.0%	58.5%
NLR	0.550	0.485-0.614	1.46	85.8%	27.7%
PLR	0.528	0.463-0.592	127.385	42.6%	69.2%
MLR	0.532	0.466-0.596	0.245	54.0%	60.0%
ESR	0.685	0.622-0.743	15.5	52.3%	76.9%
CRP	0.691	0.629-0.749	10.85	71.6%	63.1%

CAR: C-reactive protein to albumin ratio; NLR: neutrophil-lymphocyte ratio; PLR: platelet-lymphocyte ratio; MLR: monocyte-lymphocyte ratio; CRP: C-reactive protein; ESR: erythrocyte sedimentation rate; AUC: areas under the ROC curve; 95% CI: 95% confidence interval.

**Table 4:** Binary logistic regression analysis of independent predictive factors for axSpA disease activity.

Predictors	Univariate regression analyses			Multivariate regression analyses		
	*β*	OR (95% CI)	*P* value	*β*	OR (95% CI)	*P* value
Sex	0.009	1.009 (0.473-2.153)	0.982			
Age	0.023	1.023 (0.987-1.060)	0.211			
Neutrophils	0.163	1.177 (0.432-3.204)	0.750			
Lymphocytes	0.699	2.012 (0.615-6.579)	0.248			
Platelets	0.706	2.026 (0.960-4.275)	0.064			
CAR	1.665	5.287 (2.866-9.753)	<0.001	1.542	4.673 (1.423-15.348)	0.011
NLR	0.180	1.197 (0.568-2.522)	0.637	-0.316	0.729 (0.299-1.780)	0.488
PLR	0.513	1.671 (0.912-3.061)	0.097	0.013	1.013 (0.502-2.041)	0.972
MLR	0.938	2.554 (1.081-6.035)	0.033	0.613	1.845 (0.680-5.007)	0.229
ESR	1.157	3.180 (1.750-5.777)	<0.001	0.516	1.675 (0.786-3.568)	0.181
CRP	1.312	3.715 (2.044-6.754)	<0.001	-0.225	0.798 (0.239-2.663)	0.714

CAR: C-reactive protein to albumin ratio; NLR: neutrophil-lymphocyte ratio; PLR: platelet-lymphocyte ratio; MLR: monocyte-lymphocyte ratio; CRP: C-reactive protein; ESR: erythrocyte sedimentation rate; OR: odds ratio; 95% CI: 95% confidence interval.

## Data Availability

The data used to support the findings of this study are available from the corresponding author upon request.

## Supplementary Materials

Supplementary Materials Figure S1: correlations of CAR, CRP, and ESR with BASDAI and BASFI in axSpA patients.
