# Supplementary figures and images for: Correlation between C-Reactive Protein to Albumin Ratio and Disease Activity in Patients with Axial Spondyloarthritis
Source: Dis Markers. 2021 Jun 12;2021:6642486. doi: 10.1155/2021/6642486 (PMC8216814; doi:10.1155/2021/6642486)

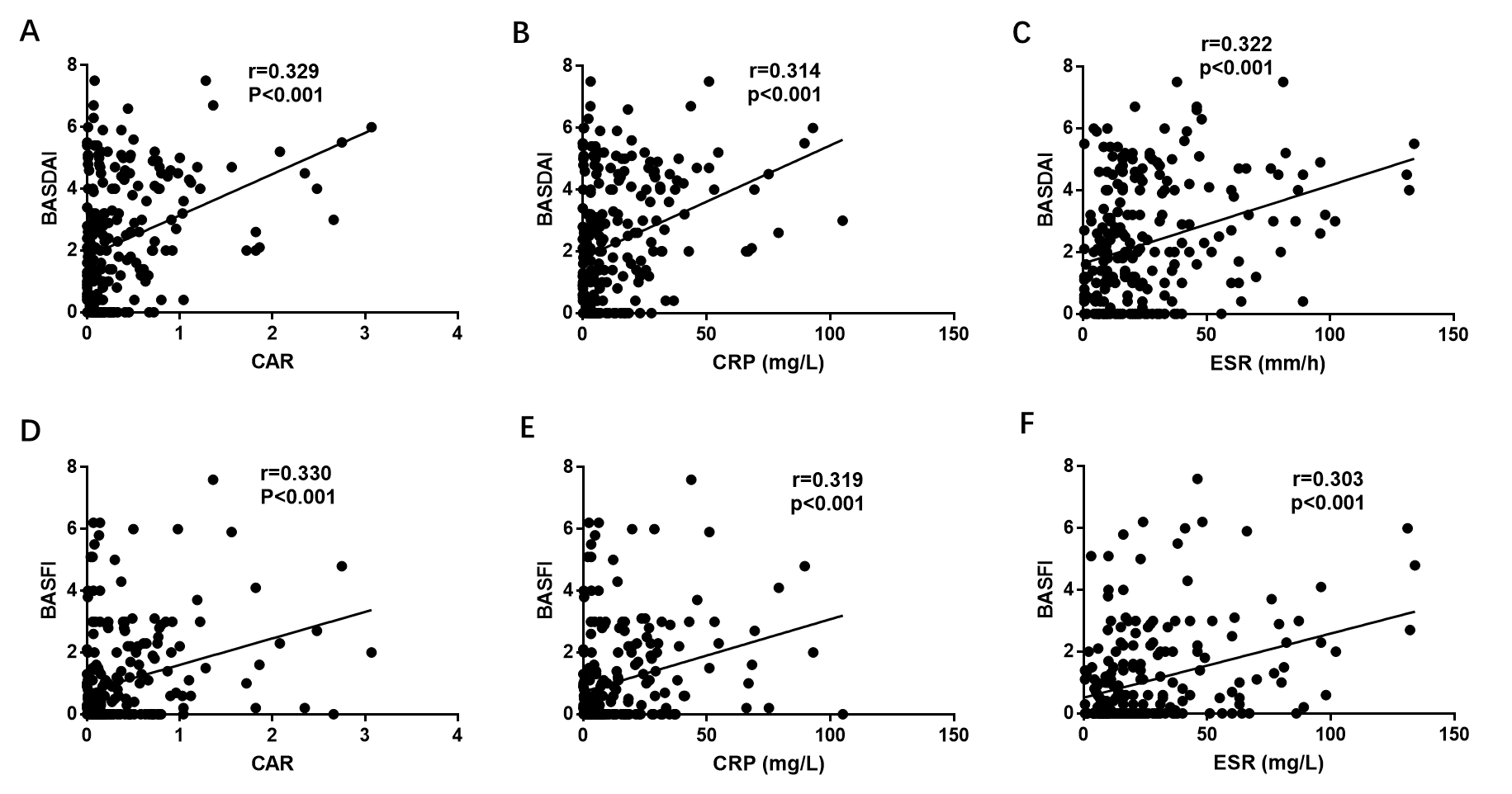


**Fig. S1 Correlations of CAR, CRP and ESR with BASDAI and BASFI in axSpA patients.**

Supplement: Supplementary Materials — Figure S1: correlations of CAR, CRP, and ESR with BASDAI and BASFI in axSpA patients. [file 6642486.f1.docx]
